# Combining indoor residual spraying with chlorfenapyr and long-lasting insecticidal bed nets for improved control of pyrethroid-resistant *Anopheles gambiae*: an experimental hut trial in Benin

**DOI:** 10.1186/1475-2875-10-343

**Published:** 2011-11-16

**Authors:** Corine Ngufor, Raphael N'Guessan, Pelagie Boko, Abibatou Odjo, Estelle Vigninou, Alex Asidi, Martin Akogbeto, Mark Rowland

**Affiliations:** 1 London School of Hygiene and Tropical Medicine, London, UK; 2 Centre de Recherches Entomologiques de Cotonou, Cotonou, Benin

**Keywords:** pyrethroid resistance, *Anopheles gambiae*, malaria control, experimental hut

## Abstract

**Background:**

Neither indoor residual spraying (IRS) nor long-lasting insecticidal nets (LLINs) are able to fully interrupt transmission in holoendemic Africa as single interventions. The combining of IRS and LLINs presents an opportunity for improved control and management of pyrethroid resistance through the simultaneous presentation of unrelated insecticides.

**Method:**

Chlorfenapyr IRS and a pyrethroid-impregnated polyester LLIN (WHO approved) were tested separately and together in experimental huts in southern Benin against pyrethroid resistant *Anopheles gambiae *and *Culex quinquefasciatus*. The bed nets were deliberately holed with either six or 80 holes to examine the effect of increasing wear and tear on protectiveness. *Anopheles gambiae *were genotyped for the *kdr *gene to assess the combination's potential to prevent the selection of pyrethroid resistance.

**Results:**

The frequency of *kdr *was 84%. The overall mortality rates of *An. gambiae *were 37% and 49% with the six-hole and 80-hole LLINs, respectively, and reached 57% with chlorfenapyr IRS. Overall mortality rates were significantly higher with the combination treatments (82-83%) than with the LLIN or IRS individual treatments. Blood feeding (mosquito biting) rates were lowest with the 6-hole LLIN (12%), intermediate with the 80-hole LLIN (32%) and highest with untreated nets (56% with the 6-hole and 54% with the 80-hole nets). Blood feeding (biting) rates and repellency of mosquitoes with the combination of LLIN and chlorfenapyr IRS showed significant improvement compared to the IRS treatment but did not differ from the LLIN treatments indicating that the LLINs were the primary agents of personal protection. The combination killed significantly higher proportions of *Cx. quinquefasciatus *(51%, 41%) than the LLIN (15%, 13%) or IRS (32%) treatments.

**Conclusion:**

The chlorfenapyr IRS component was largely responsible for controlling pyrethroid-resistant mosquitoes and the LLIN component was largely responsible for blood feeding inhibition and personal protection. Together, the combination shows potential to provide additional levels of transmission control and personal protection against pyrethroid-resistant mosquitoes, thereby justifying the additional resources required. Chlorfenapyr has potential to manage pyrethroid resistance in the context of an expanding LLIN/IRS strategy.

## Background

Long-lasting insecticidal nets (LLINs) and indoor residual spraying (IRS) are the most widely implemented methods of malaria vector control [1]. Owing to operational and logistic constraints associated with running recurrent IRS campaigns, insecticide-treated nets (ITNs) and LLINs have, until recently, been the more widely applied interventions in sub-Saharan Africa [2]. However, neither IRS nor LLINs are sufficient to achieve interruption of transmission in holoendemic areas of Africa when applied as single interventions [1].

As more resources are made available for malaria control through the Global Fund and President's Malaria Initiative, there is growing opportunity for deploying LLINs and IRS as a combination intervention [1,3]. A recent analysis of malaria control programmes which deploy both interventions together gives evidence in a range of settings of added protection among those who sleep under LLINs in IRS-treated houses [4]. The added opportunity to target malaria vectors may justify the extra cost of combination interventions. Others have raised a concern that twinned interventions may increase the selection pressure for resistance if both LLIN and IRS deploy the same insecticide [5].

The efficiency of IRS and LLINs, whether deployed singly or in combination, depends on the continued susceptibility of the vectors to the insecticides delivered through these means. Resistance to the four classes of insecticides (pyrethroids, organophosphates, organochlorines and carbamates) approved for vector control has been found in a number of *Anopheles gambiae *populations [5-8]. Pyrethroids are the ideal insecticides for treating mosquito nets owing to their knockdown effect, excito-repellent properties and low mammalian toxicity [9]. Unfortunately, pyrethroid resistance due to the *kdr *mutation is now widespread particularly in West Africa [6,8,10,11]. Reduced efficacy of LLINs and IRS due to multiple pyrethroid resistance mechanisms has been reported in Benin [6,8,12]. One of the methods used for managing insecticide resistance is to expose insect vectors to a combination of insecticides which have different modes of action. Combining of IRS and LLINs as a twinned intervention provides opportunities for resistance management as two insecticides with contrasting modes of action can be delivered at the same time and place. On a previous occasion when LLINs were combined with wall linings made of pyrethroid-treated plastic sheeting (simulating IRS) in a pyrethroid resistance area of Burkina Faso, *no *improvement on mortality of *An. gambiae *was observed over LLIN alone [13]. However, when LLINs were combined with carbamate-treated plastic sheeting on the walls, the combination proved more effective against pyrethroid resistant *An. gambiae *than LLIN alone [14,15].

Resistance to conventional insecticides and the threat of malaria control failure are the catalysts driving the development of alternative insecticides [9]. One new alternative being evaluated for vector control is chlorfenapyr, a pyrole insecticide [16,17]. Chlorfenapyr acts by targeting the oxidative pathway in insect mitochondria and shows no cross-resistance to DDT or pyrethroids [16]. The novel mode of action makes chlorfenapyr an ideal insecticide to complement the pyrethroids for the management of pyrethroid-resistant mosquitoes. Applied as IRS in experimental huts in southern Benin, chlorfenapyr at 1 g/m^2 ^induced 82.9% and 45.6% mortality among pyrethroid-resistant *An. gambiae *and *Culex quinquefasciatus *populations respectively [17]. If chlorfenapyr IRS is combined with LLINs, then mosquito vectors which fail to be killed by the pyrethroid on the LLINs, owing to resistance, can then be targeted by the chlorfenapyr treatment on the wall. A greater impact on the vector population and on transmission control would, therefore, be expected when such a combination was deployed in areas where pyrethroid-resistant *An. gambiae *or multiple vector species abound.

To test this strategy, the combination of pyrethroid LLINs and chlorfenapyr IRS was examined under experimental hut conditions in the pyrethroid-resistant area of southern Benin. The relationship between the physical integrity of the bed net material (indicated by the number of holes in the bed net) and its impact against resistant mosquitoes was also examined.

## Methods

### Study site and experimental huts

The study was carried out in experimental huts situated in Akron, a village on the periphery of Porto Novo, the administrative capital of the Republic of Benin. This is a crop production area with marshes that provide prolific breeding sites for mosquitoes over long seasons. The local *An. gambiae *is resistant to pyrethroids and DDT [12]. The nuisance mosquito *Cx. quinquefasciatus *is present year round and is resistant to pyrethroids, carbamates and organophosphate insecticides [8]. Seven experimental huts were selected for the study. These huts are typical of the West African region and are made from concrete bricks, with roofs of corrugated iron, ceilings of thick polyethylene sheeting covered with palm thatch on the interior surface and walls plastered with an unpainted cement/sand plaster. Each hut stands on a concrete base surrounded by a water-filled moat to exclude ants. Entry of mosquitoes occurs via four window slits, which are 1 cm wide and located on three sides of the hut.

### Insecticide treatments

Seven treatments were compared in the experimental huts:

1. Unsprayed hut with 6-hole, untreated bed nets

2. Unsprayed hut with 80-hole, untreated bed nets

3. LLIN with 6 holes

4. LLIN with 80 holes

5. Chlorfenapyr IRS 500 mg/m2 and LLIN with 6 holes

6. Chlorfenapyr IRS 500 mg/m2 and LLIN with 80 holes

7. Chlorfenapyr IRS 500 mg/m2

The LLIN was WHOPES approved, made of multifilament polyester fibres, factory-coated with a wash-resistant formulation of deltamethrin at a target dose of 55 mg/m^2^. The untreated bed nets were made of white 100-denier polyester multifilament net (SiamDutch Mosquito Netting Co., Bangkok, Thailand). To simulate badly worn nets, 80 holes of 2 cm^2 ^diameter were cut along each side and end panels. Nets with six holes, each measuring 4 cm^2^, two on each side and one at each end to simulate less damaged nets were also tested. Chlorfenapyr SC (BASF, 'Phantom 240SC' with 240 g chlorfenapyr/litre) was sprayed onto interior walls and plastic sheeting using a Hudson compression sprayer equipped with a flat fan nozzle. The evaluation started one week after treatment and ran for two complete rotations between June and September 2010.

### Sleepers and mosquito collection

Treatments were randomly allocated to the experimental huts. LLINs were rotated weekly between huts while the huts dedicated to the IRS treatments were fixed throughout the study as these IRS treatments could not be rotated. Seven adult men served as volunteer sleepers and were rotated between treatments on successive nights to adjust for any variation in individual attractiveness to mosquitoes. Sleepers gave informed consent and were provided with chemoprophylaxis prior to the trial. They slept in the huts from 20:00 to 05:00 each night. Mosquitoes were collected each morning at 05:00 from under bed nets, floors, walls, ceilings and verandas using aspirators and torches. The collections were transported to the laboratory where mosquitoes were identified to species and scored as blood fed or unfed and live or dead. Live mosquitoes were held in netted plastic cups and supplied with 10% honey solution. Delayed mortality was recorded at 24 h, 48 h and 72 h. Male mosquitoes were not scored.

The entomological impact of each treatment was expressed relative to the control in terms of the following:

1. Deterrence: percentage reduction in the number of mosquitoes caught in treated hut relative to the number caught in the control hut;

2. Repellency (induced exiting) due to potential irritant effect of treatments expressed as percentage of the mosquitoes collected from the veranda trap of treated hut relative to percentage caught in veranda trap of control hut;

3. Inhibition of blood feeding: reduction in blood feeding rate relative to the control. This was calculated using the following model:

100(Bf_u _- Bf_t_)/Bf_u. _where Bf_u _is the proportion of blood-fed mosquitoes in the untreated control huts and Bf_t _is the proportion of blood-fed mosquitoes in the huts with insecticide treatments;

4. Induced mortality: percentage of dead mosquitoes in treated hut at the time of collection and after a 72 h holding period relative to control hut.

The personal protective effect of the treatments which is described by a reduction in the number of blood-fed mosquitoes relative to the control hut was calculated as follows:

% Personal Protection = 100(B_u _- B_t_)/B_u _Where B_u = _is the number of blood-fed mosquitoes in the untreated control huts and B_t _is the number of blood-fed mosquitoes in the huts with insecticide treatments.

### Ethical clearance

Approval was obtained from the ethics committees of the London School of Hygiene and Tropical Medicine and the Benin Ministry of Health. Each trial participant gave written informed consent and was offered chemoprophylaxis during and for one month after the experimental hut trial.

### Molecular assays

To examine the potential for the combination treatment to prevent selection for resistance the dead and surviving *An. gambiae *were genotyped using PCR to assess the *kdr *frequency according to the method of Martinez-Torez *et al. *[18]. The resistance allele frequency at the *kdr *locus was analysed using Genepop software (version 3.3) [19].

### Data analysis

Data were entered in Excel and transferred to STATA 11.0 for further analysis. The numbers of mosquitoes collected each night were compared between treatments using a negative binomial regression model. Proportional data (exiting rate, blood feeding, and mortality) were analysed using logistic regression after adjusting for the effects of sleeper attractiveness and hut position.

## Results

Over the three-month trial, 865 *An. gambiae *sl, 7,296 *Cx. quinquefasciatus *and over l,000 *Mansonia *spp. females were caught in the huts. Only the data for *An. gambiae *and *Cx. quinquefasciatus *were analysed further.

### Anopheles gambiae

The summary results of treatment efficacy against *An. gambiae *are presented in Table 1. There was a significant difference in the number of mosquitoes collected between the seven huts (P = 0.017). These differences could be due to differences in positional attractiveness to mosquitoes or to an effect of the IRS treatments or to both. Because the IRS treatments could not be rotated it was not possible to separate the effect of hut position from IRS treatment on mosquito entry or deterrence.

**Table 1:** Summary of results obtained for *Anopheles gambiae *in the experimental huts

Hut Treatment	Untreated Net with 6 holes	Untreated Net with 80 holes	LLIN with 6 holes	LLIN with 80 holes	Chlorfenapyr IRS	Chlorfenapyr IRS + LLIN with 6 holes	Chlorfenapyr IRS + LLIN with 80 holes
**Total females caught**	**78**	**147**	**91**	**110**	**263**	**105**	**71**

**Total females dead**	**4**	**5**	**45**	**41**	**149**	**87**	**65**
**Mortality (%)**	**5.1 ^a^**	**3.4^a^**	**49.5^bc^**	**37.3^b^**	**56.7^c^**	**83^d^**	**82.3^d^**
95% Confidence Limits	0.2-10	0.5-6.3	39.2-59.7	28.2-46.3	50.7-62.6	75.7-90.1	71.4-93.3

**Total females blood fed**	**44**	**80**	**11**	**35**	**235**	**19**	**19**
**Blood feeding (%)**	**56.4 ^a^**	**54.4^a^**	**12.1^b^**	**31.8^c^**	**89.4^d^**	**18.1^bc^**	**26.8^c^**
95% Confidence Limits	45.4-67.4	45.4-62.5	5.4-18.8	23.1-40.5	85.6-93.1	10.7-25.5	16.5-37.1
Blood feeding Inhibition (%)	-	-	78	42	-	68	51
Personal protection (%)			75	56	-	57	76

**Total females in verandah trap**	**37**	**58**	**64**	**75**	**109**	**85**	**49**
**Exiting (%)**	**47.4 ^a^**	**39.5^a^**	**70.3^bc^**	**68.2^b^**	**41.4^a^**	**83^c^**	**63.4**
95% Confidence Limits	36.4-58.5	3.5-47.4	61-79.7	59.5-76.9	35.5-47.4	73.4-88.5	52.7-73.9^b^

The untreated, holed nets provided only limited protection against biting *An. gambiae*, with the proportion blood-fed reaching 54% in huts with the 80-hole nets and 56% in huts with the 6-hole nets. The difference in mosquito feeding rate between untreated 80-hole nets and untreated 6-hole nets was not significant (P = 0.532). The untreated holed nets were, however, more protective than no net at all because the proportion blood feeding in the only hut that lacked nets (chlorfenapyr IRS) was 89.4%, a value which was significantly higher than the proportion blood feeding in huts with untreated nets (P = 0.0001). The holed LLINs were more protective than holed untreated nets (P = 0.0001) due to the pyrethroid on the LLIN providing protection through its excito-repellent and knockdown effects. The LLIN with 6 holes was significantly more protective (BFI = 78%) than the LLIN with 80 holes (BFI = 42%) (P = 0.001). The addition of IRS with chlorfenapyr to a hut with LLIN did not alter the level of protection conferred by the LLIN, i.e. the proportions blood feeding with the combination were similar to that of the LLIN alone (P = 0.247 for 6-hole nets, P = 0.468 for 8-hole nets). The reduction in feeding rate with the combination was therefore attributable to the LLIN component rather than to the IRS component. The personal protection attributable to pyrethroid on the holed LLINs, relative to untreated nets, ranged from 56% to 75% protection.

The overall mortality rates with the LLIN alone ranged from 37.3% for the 80-hole to 49.5% for the 6-hole nets; the number of holes made no significant difference to the level of mortality conferred by the pyrethroid treatment (P = 0.083). The overall mortality rate with chlorfenapyr IRS treatment was 56.7%. The combination of IRS and LLIN induced overall mortality rates of 82% and 83% and this was significantly greater than the mortalities induced by LLINs alone (P = 0.0001) or by chlorfenapyr alone (P = 0.0001).

Whereas the majority of mosquitoes killed by the IRS when no LLIN was present had already blood fed (i.e. 87.2% [130/148] of the dead mosquitoes had blood-fed beforehand), only a minority of dead mosquitoes had managed to blood feed when a LLIN was present in the IRS treated hut (i.e. 9.2% [8/87] of the dead mosquitoes had blood fed beforehand through the 6-hole nets and 26.3% [17/65] through the 80-hole nets). This indicates that in the absence of a LLIN the mosquitoes will blood feed on the sleeper before alighting on the insecticidal wall. But in the presence of an LLIN many mosquitoes alight on the wall and are killed before managing to feed.

### Culex quinquefasciatus

The effects of the treatments on *Cx. quinquefasciatus *are presented in Table 2. Untreated nets with 80 holes were less protective against *Cx. quinquefasciatus *than untreated nets with six holes (P = 0.0001). In the absence of any net a higher proportion of mosquitoes were able to blood feed (77% managed to blood feed in the huts with chlorfenapyr IRS and no net), indicating that the holed net was a partial barrier to *Culex *biting and feeding. The pyrethroid on the LLIN was highly protective and the level of blood feeding inhibition by the insecticide was higher for *Cx. quinquefasciatus *than for *An. gambiae*. However, only a small proportion of *Cx. quinquefasciatus *were killed by the pyrethroid (~15%), and this proportion was smaller than the proportion of *An. gambiae *killed by the same treatment. Chlorfenapyr IRS killed 32.3% of *Cx. quinquefasciatus*. The combination of IRS and LLIN was additive, killing in the range of 40-50%. As was observed with *An. gambiae*, the combination was protective against *Cx. quinquefasciatus *(mostly due to the LLIN component) and also succeeded in killing many *Cx. quinquefasciatus *(mostly due to the IRS component). As with *An. gambiae*, the proportion of *Cx. quinquefasciatus *blood feeding was dependent on the number of holes in the LLIN, irrespective of whether chlorfenapyr IRS was present or not.

**Table 2:** Summary of results obtained for *Culex quinquefasciatus *in the experimental huts

Hut Treatment	Untreated Net with 6 holes	Untreated Net with 80 holes	LLIN with 6 holes	LLIN with 80 holes	Chlorfenapyr IRS	Chlorfenapyr IRS + LLIN with 6 holes	Chlorfenapyr IRS + LLIN with 80 holes
**Total females caught**	**533**	**1370**	**1014**	**1018**	**1507**	**1260**	**1083**

**Total females dead**	**9**	**27**	**152**	**133**	**487**	**642**	**455**
**Mortality (%)**	**1.7^a^**	**2.0^a^**	**15.0^b^**	**13.1^b^**	**32.3^c^**	**51.0^d^**	**42.0^e^**
95% Confidence Limits	0.6-2.8	1.2-2.7	12.8-17.2	11.0-15.1	30.0-34.7	48.2-53.7	39.1-45.0

**Total females blood fed**	**180**	**666**	**55**	**124**	**1157**	**52**	**107**
**Blood feeding (%)**	**33.8^a^**	**48.6**^b^	**5.4**^c^	**12.2**^d^	**76.8^e^**	**4.1**^c^	**9.9^d^**
95% Confidence Limits	29.8-37.8	46.0-51.3	4.0-6.8	10.2-14.2	74.6-78.9	3.0-5.2	8.1-11.7
Blood feeding Inhibition (%)	-	-	84	75	-	88	80
Personal protection (%)	-	-	-	81	-	71	84

**Total females in verandah trap**	**225**	**389**	**677**	**603**	**638**	**784**	**687**
**Exiting (%)**	**42.2 ^a^**	**28.4^b^**	**66.8^c^**	**59.2^d^**	**42.3^a^**	**62.2^d^**	**63. 4^cd^**
95% Confidence Limits	38 - 46.4	26 - 30.8	63.9 - 70	56.2 - 62.2	40 - 45	60 - 65	60.8 - 66.3

Numbers in the same row sharing the same superscript do not differ significantly (*P *≥ 0.05)

#### Genotype selection

The results for the molecular studies are presented in Table 3. For each treatment, the frequency of the *kdr *allele did not differ between the survivor and dead collections of *An. gambiae *at the 5% significance level. These results did not show any selective advantage to *kdr *in the presence of the LLIN. Nor did it show any selective neutrality or disadvantage to *kdr *in the presence of the combination treatment. Initial genotyping of 100 adult *An. gambiae*, which emerged from larvae collected at the field site (Akron), showed a *kdr *frequency of 0.91, a value consistent with the samples collected from the huts. This high frequency of *kdr *and the relatively small samples analysed made it impossible for the current study to demonstrate any differential selection of *kdr *between the LLIN and combination treatments.

**Table 3:** *Kdr *allelic frequency (*kdr *alleles/total *kdr *and susceptibility alleles) among live and dead *Anopheles gambiae*

Treatments	Alive	Dead
Chlorfenapyr (500 mg/m^2^) IRS	**0.77 (14/18)**	**0.84 (32/38)**
Chlorfenapyr (500 mg/m^2^) IRS + LLIN with 80 holes	**1.0 (25/25)**	**0.90 (18/20)**
LLIN with 80 holes	**0.82 (33/40)**	**0.86 (19/22)**
Untreated net with 80 holes	**0.82 (41/50)**	**0.89 (16/18)**

## Discussion

A combination of IRS and LLINs can only be justified economically if it provides greater levels of protection or greater transmission control than is achievable by the single interventions. The present comparisons show that the combining of chlorfenapyr IRS and pyrethroid LLINs in the same hut provides personal protection (mostly attributable to the LLIN component) and transmission control potential (mostly but not wholly attributable to mortality induced by IRS) over and above what the individual components are able to achieve. With the massive injection of international aid by the Global Fund and President's Malaria Initiative for universal coverage of LLINs and IRS transmission control, the roll-out of such a combination intervention appears well justified on the basis of the present suggestive small scale study.

The spectre of resistance haunts our capacity to control malaria in the future. Pyrethroid resistance due to *kdr *or metabolic mechanisms is springing up in countries across sub-Saharan Africa [5]. While clear demonstration that such resistance is impacting negatively on control has yet to be made, there is growing evidence that pyrethroid-treated ITNs and LLINs provide less protection in areas such as southern Benin where multiple pyrethroid resistance has become prevalent [6,8]. Confronted with resistance, LLINs start to lose their protectiveness once they become holed and the more holes they accrue the less protective they become, as demonstrated in the present study.

Previously it was shown that chlorfenapyr is capable of controlling pyrethroid-resistant *An. gambiae *and *Cx. quinquefasciatus *when applied as IRS [17]. The most important new findings from the current study were the additive levels of mortality and the reduced levels of blood feeding that can be achieved when chlorfenapyr IRS is combined with pyrethroid LLIN. A mortality of ~80% is similar to what can be achieved with pyrethroid IRS in an area of full susceptibility [6]. Taken together with the partial protection still to be had from LLINs, the combination of LLINs and chlorfenapyr IRS may prove to be a route out of the predicament presented by pyrethroid resistance.

These findings stand in contrast to the experimental hut studies in Burkina Faso in which a combination of pyrethroid-treated wall linings and ITN failed to induce any increase in mortality of malaria vectors over that of ITN alone [13]. This difference can be attributed to the fact that the vector population in the current study is fully susceptible to the active component (chlorfenapyr) in the IRS treatment. These results corroborate previous experimental hut studies in Benin and in Burkina Faso where pyrethroid-treated nets were combined with carbamate-treated plastic wall sheetings to which there was also little or no resistance [15].

The relatively high mortality of mosquitoes in chlorfenapyr IRS-treated huts confirms the potential of the pyrole insecticide as an alternative IRS treatment [17]. Hut trials of IRS, whether with pyrethroid, carbamate, OP or pyrole [6,17,20], show little or no evidence of blood feeding inhibition among the mosquitoes collected from the huts. The inference is that hut-entering mosquitoes approach, contact and feed upon the host before resting on the insecticide-treated walls where they then pick up a lethal dose. The current trial supports that inference. But when faced with a barrier presented by the LLIN, some mosquitoes succeed in penetrating the holes and feed, while others fly away unfed from the net, not to leave the hut but to alight on the walls where they then pick up a lethal dose of chlorfenapyr before resuming host-seeking flights. This train of events could explain the higher proportion of unfed, dead mosquitoes in the combination LLIN/IRS huts than in the single intervention LLIN or IRS huts. It is perhaps the to-ing and fro-ing between bed net and wall that results in higher pick-up of insecticide and higher mortality rates than is achieved by blood-fed mosquitoes that after feeding on the host simply alight on the treated wall and remain stationary.

*Cx. quinquefasciatus*, the nuisance mosquito in urban West Africa and filariasis vector in East Africa, is strongly resistant to pyrethroids and consistently records low mortality rates (less than 15%) in the presence of LLINs in experimental huts studies [21]. This is due to multiple resistance mechanisms to pyrethroids and organophosphates [8]. In the current study, mortality of *Cx. quinquefasciatus *increased to 51% when the LLINs were combined with chlorfenapyr IRS. The fact that the combination killed up to three times more *Cx. quinquefasciatus *mosquitoes than LLINs alone enhances the acceptability and compliance of this combined strategy for malaria control in peri-urban settings.

## Conclusion

Combining chlorfenapyr IRS and LLINs has an additive effect on the mortality of pyrethroid-resistant mosquitoes. In areas of high pyrethroid resistance or high transmission intensity, control programmes with sufficient resources should consider implementing a combination intervention of LLIN plus non pyrethroid-based IRS. Chlorfenapyr IRS is shown to be an ideal supplement to pyrethroid LLIN for improving the control of pyrethroid-resistant mosquitoes.

## Competing interests

The authors declare that they have no competing interests.

## Authors' contributions

CN: Project supervision, data entry, data analysis and interpretation, drafted the manuscript. RN: Project design and supervision, data interpretation, manuscript revision. PB, AO, EV: Conducted the field trials, molecular analyses, bioassay testing. AA: Co-supervision, data processing, manuscript revision. MA: Co-design of the project, manuscript review and revision. MR: Project design, coordination with IVCC and BASF, interpretation of the results, revision of the manuscript. All authors read and approved the final manuscript.
